# Verification of Fatigue Damage and Prognosis Related to Degradation of Polymer-Ceramic

**DOI:** 10.3390/ma14185147

**Published:** 2021-09-08

**Authors:** Piotr Kosiński, Piotr Żach

**Affiliations:** Faculty of Automotive and Construction Machinery, Warsaw University of Technology, 02-524 Warsaw, Poland; piotr.zach@pw.edu.pl

**Keywords:** laminated windshield, pedestrian safety, glass failure, polyvinyl butyral, temperature effect

## Abstract

Statistically, road accidents involving pedestrians occur in the autumn and winter months, when outdoor temperatures reach −30 °C. The research presented in this paper investigates the impact of a pedestrian’s head on laminated windscreen, taking into account the effects of external temperature, heating of the windscreen from the inside, and fatigue of the glass. The automotive laminated windscreen under study is made from two layers of glass and a Polyvinyl Butyral (PVB) resin bonding them together. PVB significantly changes its properties with temperature. The Finite Element Method (FEM) simulations of a pedestrian’s head hitting the windscreen of an Opel Astra II at <−30 °C, +20 °C> were performed. The obtained Head Injury Criterion (HIC) results revealed an almost twofold decrease in safety between +20 °C and −20 °C. The same test was then performed taking into account the heating of the windscreen from the inside and the fatigue of the glass layers. Surprisingly, the highest HIC value of all the cases studied was obtained at −30 °C and heating the windscreen. The nature of safety changes with temperature variation is different for the cases of heating, non-heating, and fatigue of glass layers. Glass fatigue increases pedestrian safety throughout the temperature range analysed.

## 1. Introduction

The safety of so-called Vulnerable Road Users (VRU), i.e., pedestrians, cyclists, motorcyclists, and all those who are potential victims during a collision with a motor vehicle, is currently one of the leading topics in the field of vehicle safety. In 2007, pedestrian safety tests were made compulsory worldwide and in the European Union [1] for the homologation of motor vehicles. That action was the genesis of the VRU safety improvement process. A line of work has been set out, which includes improvement of safety not only for users inside the vehicle, but also for those outside. Tests for pedestrian safety on the vehicle are carried out for fixed conditions: temperature of 20 ± 4 °C and humidity of 40 ± 30% [2].

One of tests of this type is when a pedestrian’s head strikes the windscreen of a car [2]. The accidents involving VRU occur in a variety of climatic conditions. The windscreen operating temperature is affected by both the outside temperature, external factors such as wind, vehicle speed, precipitation, the vehicle’s internal temperature, and the consequences of the vehicle’s interior heating system operation.

A car windscreen is a laminated panel usually made up of two layers of glass with Polyvinyl Butyral (PVB) resin bonding them together. The issues of verification of phenomena for laminated sandwich systems with the use of two-dimensional and solid elements have been the subject of scientific studies [3,4,5,6,7,8,9]. The laminate behaviour was investigated by Timmel [10] using 2D shell elements with smeared technique. This type of modelling uses two layers of coincident shell elements with the same thickness which represents all layers of composite by calculate thickness and density equivalence. The assumption is full bonding between glass layers and PVB, which can be adjusted by stiffness factor, but always need correlation and are used only for a specific type of load case. Later work by Peng [11] consider additionally laminate windshield modelling as three separate layers of shell element with tie contacts or with share nodes.

In 2002 D’Haene [12] described experimentally the relation of shear modulus of PVB for different temperatures. The data shows a 10-foold increase of the modulus between +25 °C and −5 °C. The range between −5 °C and +25 °C do not cover the VRU and passenger cars daily usage environment. Furthermore, the investigation of pedestrian safety in any different condition than standard +20 °C was not considered in the science.

Other studies (e.g., Jun Hu [13]) were focused on comparison between low speed and high speed impact scenarios of PVB and on compression experiments on PVB material. Another interesting study by Gevers investigated optimization of laminated windshields [14]. This was enhanced work performed by a car manufacturer where they improved the correlation between test and laminated windshield simulation models from 2008 to 2011. The latest model matches closely with the real impact of a headform. The assessment of the influence of the boundary conditions on the delamination process of layered glass structures was described in [4,15]. The issues of elasticity solution and the influence of the thickness of laminated glass structural elements are discussed in [5,16].

Based on the author study, the mechanical properties of PVB were found to be strongly temperature dependent. The PVB tensile tests for temperatures from −30 °C up to +20 °C are presented in the author’s publication [17]. It describes the issue of fatigue in the polymer–ceramic system, which has not been analysed so far, using the example of a laminated glass panel subjected to loads from external temperatures <−30 °C, 0 °C> and forced heating of the panel from the inside. The effect of fatigue of the laminated windscreen associated with car body torsion on the behaviour of the system was also analysed.

## 2. Materials and Methods

The validity of research related to pedestrian safety at temperatures much lower than +20 °C is reflected in the statistics of accidents involving pedestrians in Poland in 2019, prepared by the Police Headquarters [18].

The highest number of accidents involving pedestrians was recorded in the months from October to January. The highest number of such accidents was recorded in December, i.e., 13.8%. In November it was 10.9%. The number of injured and killed is also highest during the autumn and winter months. The highest number of killed was recorded in December, i.e., 16%. In January it was 12%. The highest number of injured was recorded in December, i.e., 13.7%, and in November 11.1%. The pedestrian fatal injuries are mainly related to head injuries during the collision. It is necessary to carry out a verification of the problem of pedestrian safety in various thermal conditions, with particular attention paid to the temperatures prevailing in the autumn and winter months.

The issues of pedestrian head impact on laminated windscreen during an accident involving VRU for operating conditions covered by the temperature range <−30 °C, +20 °C> have been analysed. The following cases have been characterised:

(1) collision with VRU at a laminated windscreen temperature of −30 °C, all vehicle heating systems were switched off;
(2) collision with VRU at a laminated windscreen temperature close to −30 °C, the vehicle heating systems were on during vehicle start-up but the windscreen was not fully heated;
(3) collision with VRU at a laminated windscreen temperature close to −30 °C, the vehicle heating systems were on during vehicle start-up but the windscreen was not fully heated, the laminated windscreen subjected to additional fatigue loads from under-window frame operation related to the vehicle body torsional rigidity.

**Figure 1:**
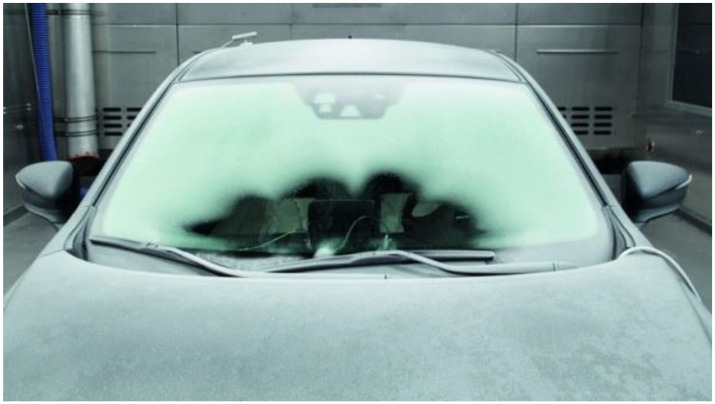
Windscreen heating.

When considering cases 2 and 3, attention should be paid to the complex state of operational loads to which the laminated vehicle windscreen is subjected when operating at temperatures characteristic for glass transition of the PVB polymer bonding layer. The additional factors influencing temperature reduction and non-uniform heating of the windscreen include: the derivative of vehicle speed and resistance to motion, the effect of wind, precipitation. Figure 1 shows the consequences of blowing warm air (heating) on the windscreen of a vehicle at negative environment temperature.

The windscreen shown in Figure 1 is heated from the inside, at the bottom. Blowing of warm air onto the windscreen creates the distribution of thermal zones. For a given moment in time, we can determine the equilibrium state of the heated and unheated zones. The heated zone forms a characteristic area with an edge shaped like four segments of a circle. The phenomenon of heat wave propagation is symmetrical with respect to the plane of symmetry of the vehicle.

### 2.1. Pedestrian Head Impact Model

**Figure 2:**
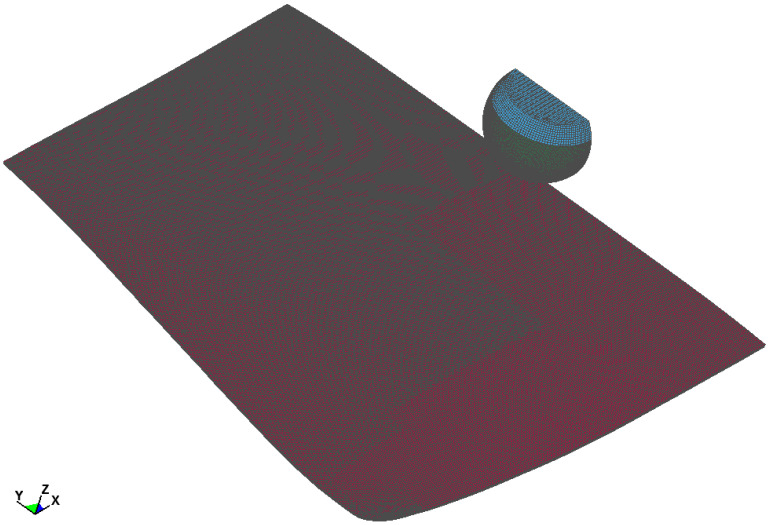
Strike of head impactor on the laminated windscreen—symmetrical model.

To investigate the phenomena described in Section 2, the pedestrian head impactor test [2] was used. During the test, a head impactor of a mass of 4.8 kg strikes the rigid windscreen at a speed of 35 km/h (Figure 2). The windscreen model was constructed upon the basis of a 3D scan of the real car windscreen conducted by the Automotive Industry Institute in Warsaw. The adult head impactor used was certified and is described in the publication [18]. The velocity vector of the impactor was perpendicular to the windscreen at the point of impact. The glass was restrained at the edges, where three displacement degrees of freedom were removed. The case of an impact at the central point of the laminated windscreen was taken for analysis. The whole of the model was simplified using symmetry of boundary condition, shape, and loading to speed up the analysis. Contact between the Impactor was modelled used *Surface_to_surface contact with SOFT = 2, SOBT = 3 and Depth = 5. The static and dynamic friction was defined ($\mu_{s}$ = 0.3, $\mu_{d}$ = 0.25). The analysis was done in LS-DYNA solver.

The windscreen model consists of two layers of glass 2.1 mm thick and a PVB layer 0.76 mm thick. Based on the results of works [17,19], the polymer–ceramic structure was described by solid elements: tetrahedral for the case of glass (four nodes with one integration point), pentahedral for the case of PVB. Average size of windshield element was 1 mm. The impactor foam was meshed by 10 mm elements and impactor core element size was 10 mm as well.

#### 2.1.1. Glass Material Model

The glass material model represented the Johnson–Holmquist material model. That model has a failure criterion based upon the Hugoniot condition. It describes the behaviour of a solid during the sudden action of an external forcing. The Johnson–Holmquist model is described by the following equations.

Stress equation:

(1) $$\sigma_{ij}=-p\left(\varepsilon_{kk}\right)\delta_{ij}+2\mu \varepsilon_{ij}$$

where: $p\left(\varepsilon_{kk}\right)$—state equation, $\delta_{ij}$—Kronecker delta, $\varepsilon_{ij}$—strain, μ—shear modulus, $\varepsilon_{kk}$—shear coefficient.

State equation:

(2) $$p\left(\varepsilon_{kk}\right)=p\;\left(\frac{\rho }{\rho_{o}}-1\right)$$

where: ρ—current density, $\rho_{o}$—initial density.

The Hugoniot limit stresses are expressed by the equation:

(3) $$\sigma_{h}=p_{HEL}\left(\rho \right)+\frac{2}{3}\sigma_{HEL}\left(\rho ,\mu \right)$$

where: $p_{HEL}$—Hugoniot elastic limit pressure, $\sigma_{HEL}$—Hugoniot elastic limit stress.

The stresses before failure are described by the following equation:

(4) $$\sigma_{intact}^{*}=A{\left(p^{*}+T^{*}\right)}^{n}\left[1+Cln\left(\frac{d\varepsilon_{p}}{dt}\right)\right]$$

where: *A*, *C*, *n*—material constants, *t*—time, $\varepsilon_{p\;}$—inelastic strain, $\sigma^{*}=\frac{\sigma }{\sigma_{HEL}};p^{*}=\frac{p}{p_{HEL}};T^{*}=\frac{T}{\sigma_{HEL}}$—normalized stress, pressure, and tensile strength.

The uniaxial stresses of complete fracture are represented by equation:

(5) $$\sigma_{fracture}^{*}=B{\left(p^{*}\right)}^{m}\left[1+Cln\left(\frac{d\varepsilon_{p}}{dt}\right)\right]$$

where: *B*, *C*, *m*—material constants.

The current stresses are calculated according to the following equation:

(6) $$\sigma^{*}=\sigma^{*}{}_{initial}-D\left(\sigma^{*}{}_{initial}-\sigma^{*}{}_{fracture}\right)$$

where: *D*—damage accumulation factor.

The evolution of failure is described by the equation:

(7) $$\frac{dD}{dt}=\frac{1}{\varepsilon_{f\;}}\frac{d\varepsilon_{p\;}}{dt}$$

and the deformation of failure is described by the formula:

(8) $$\varepsilon_{f\;}=D_{1}{\left(p^{*}+T^{*}\right)}^{D_{2}}$$

where: $D_{1},\;D_{2}$—material constants.

Characteristics of experimental laminated glass works described in articles [3,20,21]. The application of presented model in Finite Element Method simulations required using the material data which were adopted from [22] and based upon the results of the PVB polymer own work described in Section 2.1.2.

#### 2.1.2. PVB Material Model

**Figure 3:**
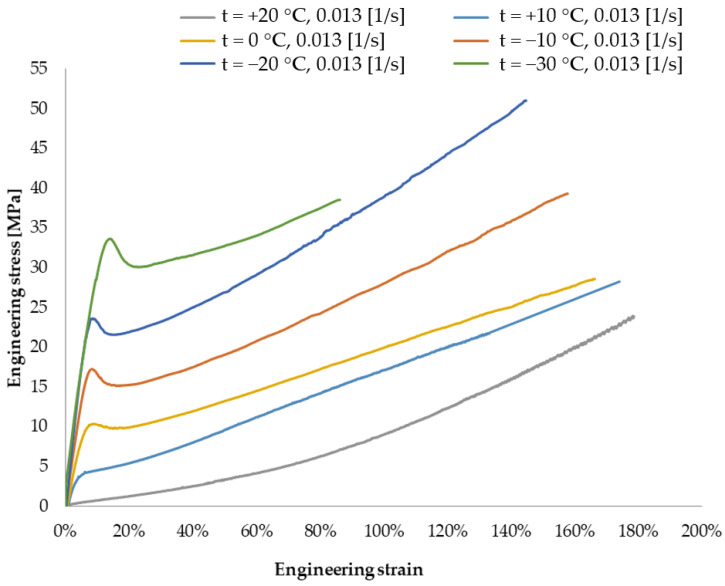
PVB tensile curves—*t* = <−30, +20> °C for 0.013 [1/s].

The characteristics of the polyvinyl butyral resin are described in [23,24,25,26,27]. Due to the non-unequivocal replication of PVB features required to perform structural identification physical and mechanical properties of PVB material, the experimental testing of the polymer has been carried out in accordance with PN-EN ISO 527:1988 standard “Plastics—Determination of mechanical properties at static tension—Test conditions for films and plates” [28] for the temperature range: −30 °C to +20 °C and a strain rate in the range of 0.065 to 0.013 [1/s]. The specimen was staying at the specified temperature 20 min. before loading. Figure 3 shows the stress–strain curves developed upon the results for the analysed temperatures and strain rate of 0.013 [1/s].

**Figure 4:**
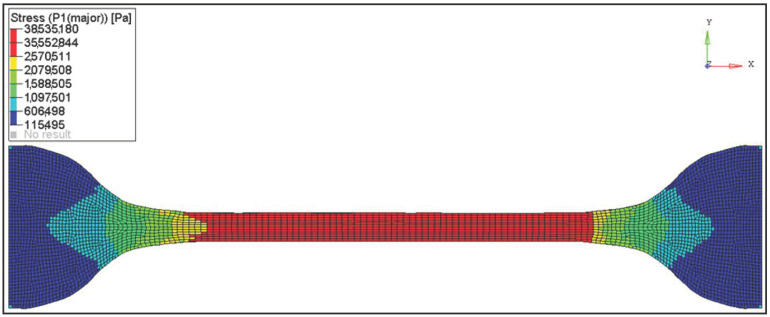
PVB tensile test—FEM at *t* = −30 °C.

**Figure 5:**
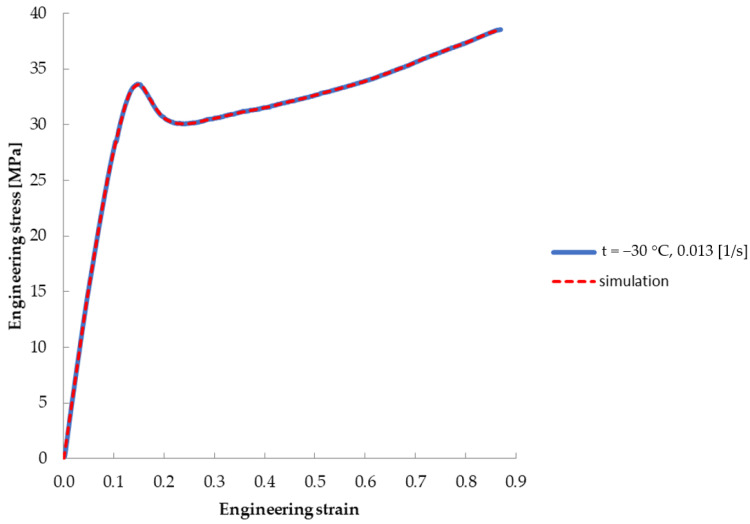
Comparison of test curve and FEM simulation curve (*t* = −30 °C).

The tensile tests have been performed in a virtual environment for an analogous polymer operation range. A Marlow material model has been used to describe the operation of polyvinyl butyral. The engineering stress–strain curve obtained from the simulation was compared with the curve obtained from material tests. The result of the analyses performed using the Finite Element Method (FEM) is presented in Figure 4. Figure 5 shows a comparison of the stress–strain relationship obtained in the tensile test for PVB at *t* = −30 °C: actual and simulation.

It has been proven that the PVB behaviour changes with the temperature change. The stress–strain relationship for the temperature of +20 °C was described by a polynomial of the second order, for the temperature range of +10 to −30 °C by a polynomial of the third order, while the influence of the first extremum increased with decreasing temperature.

A very good level of convergence of the solution has been obtained. The Marlow model has been proven to be adequate to describe the material behaviour with such unique course as the revealed one. Used Marlow model allowed to achieve a convergence despite the non-linearity of the curve over the entire range of analysed temperatures.

## 3. Simulation of Head Impact on the Laminated Windscreen in Varied Temperature Range

The simulations of head impact on the laminated windscreen have been performed for operation temperatures of +20 to −30 °C without taking the influence of the additional energy stream coming from the heating system into account. The numerical description of the modelling process was based on the information contained in the publications [10,29,30,31,32]. Sequentially for the temperature range of 30–0 °C the course of phenomena occurring during the impact has been analysed, taking into account the influence of local changes in the polymer structure under the influence of air blow coming from the windscreen heating system. The influence of fatigue loads affecting the operational character of the laminated windscreen from torsional deformations of the car body has been considered. It has been assumed that the properties of glass do not change with temperature which was discussed in detail in the articles [33,34,35,36,37]. The temperature-dependent behaviour of PVB was taken into account.

The criterion for assessing the pedestrian safety is the Head Injury Criterion (HIC) described by the relation (9):

(9) $$HIC=\max\{{\left[\frac{1}{t_{2}-t_{1}}\overset{t_{2}}{\underset{t_{1}}{\int }}adt\right]}^{2.5}\left(t_{2}-t_{1}\right)\}$$

where: *a*—head impactor acceleration, $t_{2},\;t_{1}$—measurements start and end moments in time.

It is desirable that the levels of HIC values are as small as possible. The measurable consequences of the analysed solution are: reduction of head injuries and increase of VRU safety. During calculation of the maximum HIC value the time intervals *t*_2_ − *t*_1_ below 15 ms are omitted. The lower the maximum HIC value, the smaller the head injury and better VRU safety.

**Figure 6:**
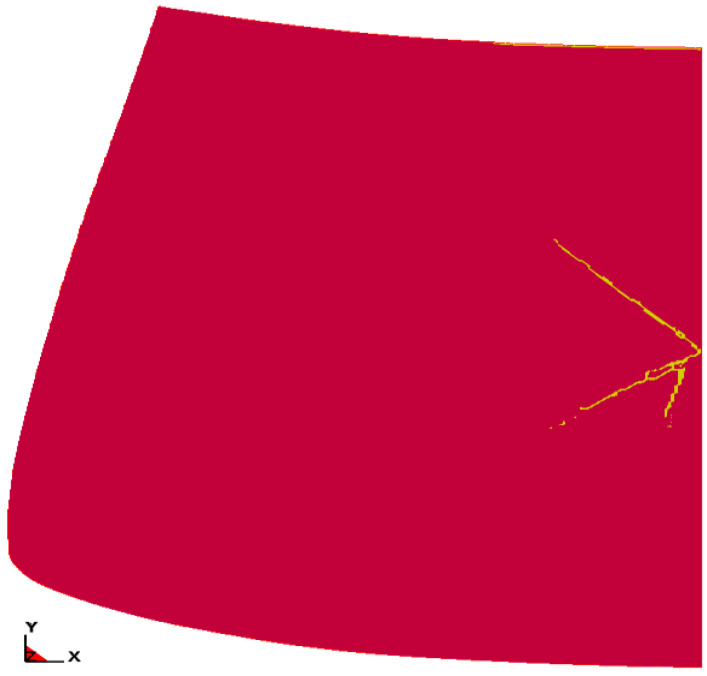
Results of simulation of head impactor impact on the windscreen of an Opel Astra—temp. = −30 °C (*t* = 21 ms).

Figure 6 shows the results of the pedestrian head impactor simulation for temperature of −30 °C.

**Figure 7:**
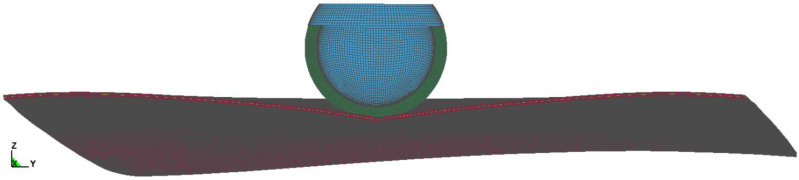
Maximum windscreen strain—temp. = −30 °C (*t* = 8 ms).

**Figure 8:**
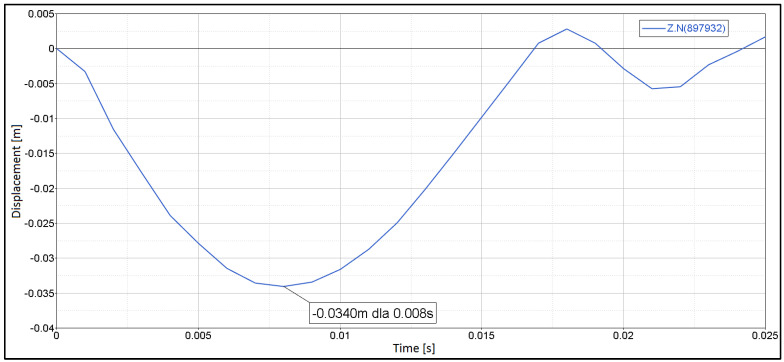
Displacement of windscreen int the point of impactor impact—temp. = −30 °C (*t* = 8 ms).

It has been found that the maximum displacement of the laminated windscreen at the impactor impact point in the Z-axis direction was x = 34.0 mm for *t* = 8 ms. The results are shown in Figure 7. The nature of changes in displacement over time for the case analysed is shown in Figure 8.

**Figure 9:**
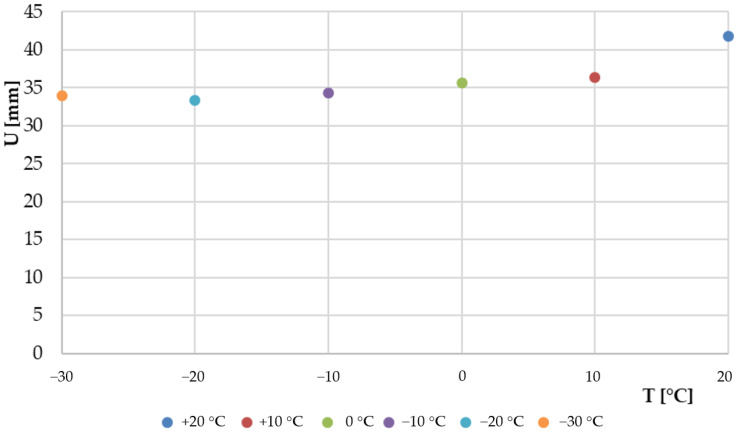
The maximum displacement of laminated windscreen due to head impactor impact as a function of temperature.

A summary of the results of the maximum windscreen displacement in the Z-axis direction resulting from the head impactor impact, for the temperatures analysed, is shown in Figure 9.

After analysis of verified cases it has been proven that the susceptibility of laminated windscreen with a PVB membrane decreases with decreasing temperature which is in line with [38]. The windscreen displacement resulting from the head impactor impact was: 41.8 mm for temp. of +20 °C, 33.4 mm for temp. of −20 °C. For temperature of −30 °C the maximum displacement was 34.0 mm. The displacement value is slightly higher than for −20 °C. It is a derivative of changes found in the PVB tension process for the temperature range of −20 to −30 °C, which was reflected in the locally varying glass cracking process.

**Figure 10:**
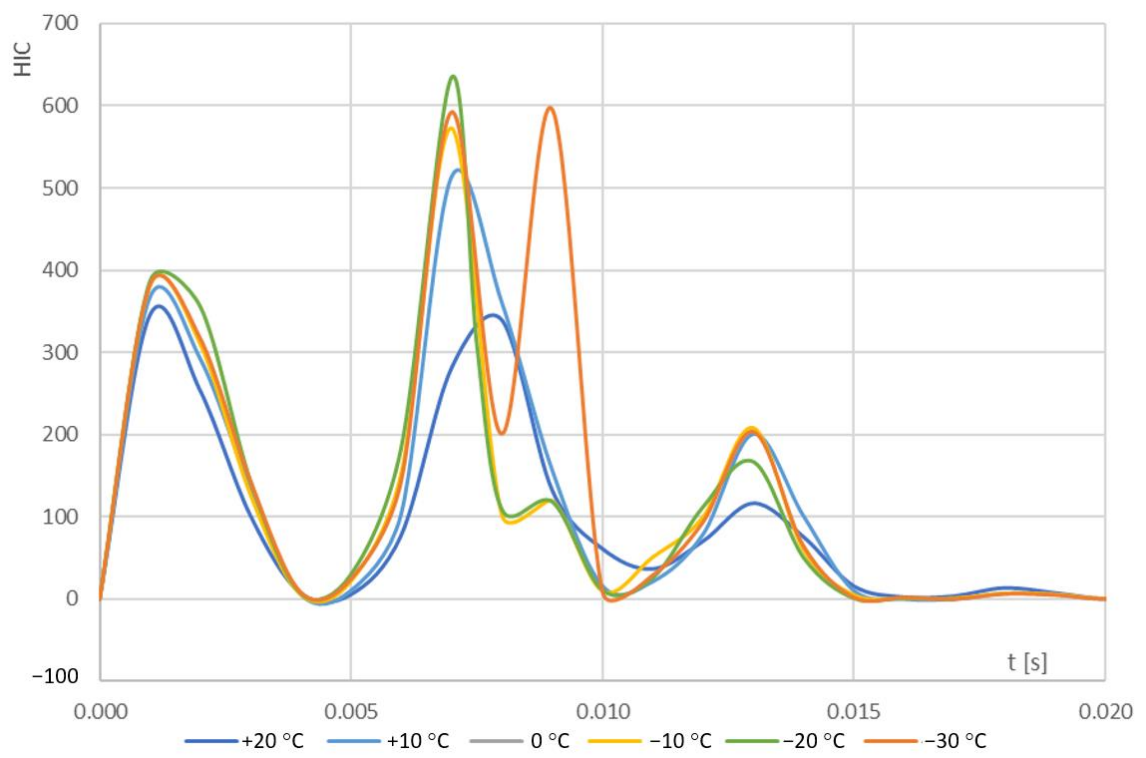
HIC depending on temperature.

Figure 10 shows the nature of HIC changes obtained from the numerical simulations for the temperature ranges considered.

The maximum HIC values for the temperature ranges considered are shown in Table 1.

**Figure 11:**
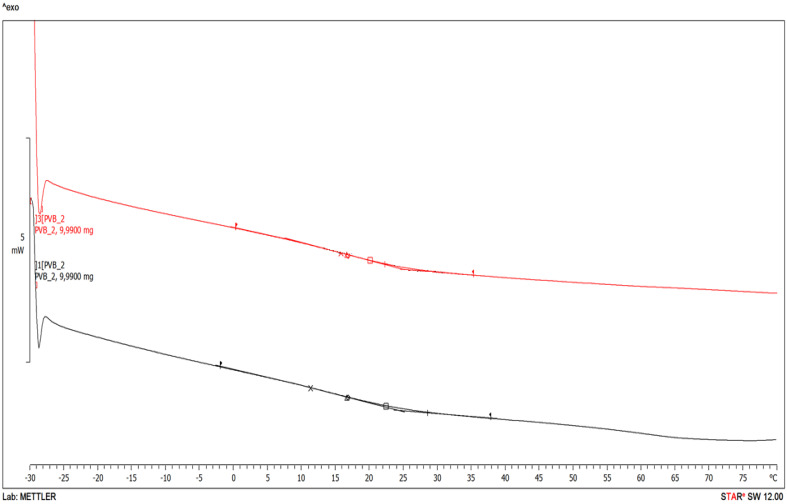
PVB DSC curves.

The maximum value for the head injury criterion (HIC) has been obtained for temperature of −20 °C. For all analysed cases of HIC change over time, excluding the temperature of −30 °C, a single extremum was found. For the temperature of −30 °C two extrema have been determined. The phenomenon is related to the nature of changes in the polymer structure, resulting from the transition of PVB to the vitreous—brittle state, which was confirmed by the results of the differential scanning calorimetry (DSC) tests performed by the authors. Figure 11 and Table 2 show the character of the structural changes of the PVB bonding shell as a function of temperature.

The representation of the phenomena in the test samples was very high stress value for the first maximum, as shown in Figure 5. An increase in stress level was observed up to 33.6 [MPa]. Based on analyses, it has been proven that the most serious pedestrian head injuries are to be expected following an accident that would occur in the temperature range of −20 °C and −30 °C.

Comparing the HIC values at +20 °C—the reference level for which vehicle tests related to pedestrian safety are performed—with the results obtained for tests in range of −30 °C < *t* < 20 °C, a very large increase in the HIC values was observed for +10 °C. Moreover, as temperatures drop throughout the year, there is an increase in conditions that adversely affect the course of accidents, i.e., reduced visibility, rain, snow, and fog. There are also pedestrian visibility restrictions. The above-mentioned factors increase the number of accidents involving VRU.

## 4. Thermo-Mechanical Fatigue Loads of Laminated Windscreen versus the HIC Criterion

### 4.1. Heating of the Windscreen

**Figure 12:**
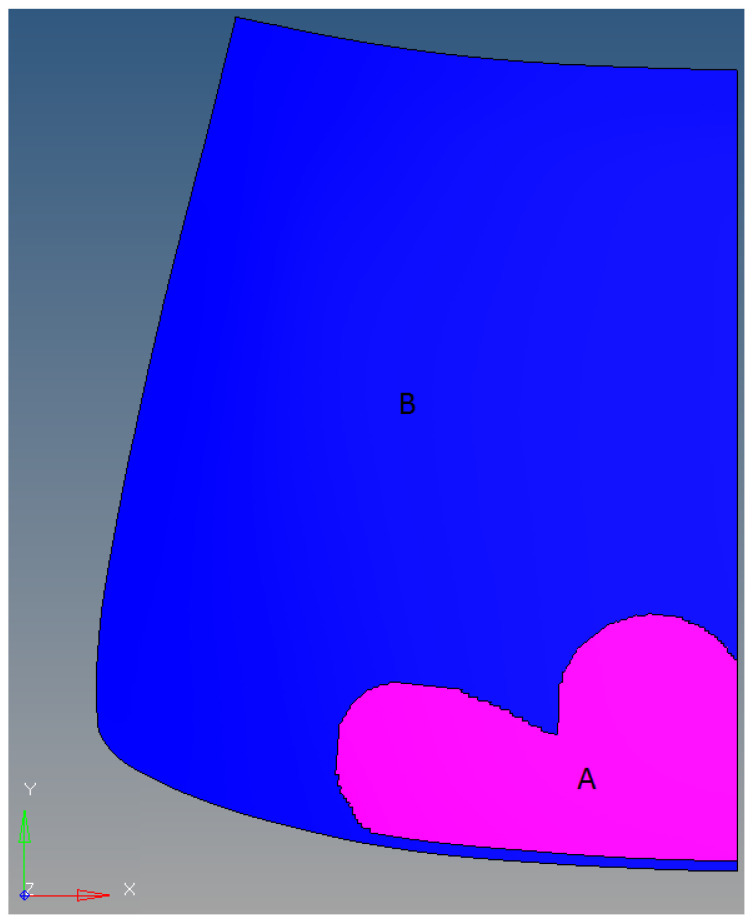
Division zones of PVB material ((**A**)—heated zone, (**B**)—unheated zone).

The analyses of phenomena occurring during the impact of a pedestrian’s head on laminated windscreen in the temperature range of <−30 °C, 0 °C> have been carried out, taking into account the blow heating of the windscreen. The phenomena occurring in the structure of laminated windscreen at negative temperatures, subjected to the influence of a stream of warm air, have been analysed. Based on the heat wave propagation diagram shown in Figure 1, a thermal load model of a laminated windscreen shown in Figure 12 has been prepared. The experiments described in Section 2.1 for the construction of numerical structure of PVB in the laminated windscreen were used to prepare the model and as well the information from the references [39,40].

The amount of heat applied from the heating system to the windscreen (zone A marked in Figure 12) was assumed to be constant which is in line with the recommendations made in the articles [41,42]. The temperature gradient arising between the inner glass layer and the PVB layer was calculated from relation (10).

(10) $$Q_{const}=\lambda \frac{S\Delta T_{const}t}{d}$$

where: $Q_{const}$—amount of heat flowing through the body, λ—thermal conductivity coefficient, *S*—cross-sectional area through which heat flows, *t*—flow time, $\Delta T_{const}$—temperature difference in direction of heat conduction, *d*—thickness of the body through which heat flows.

A gradient value of 20 °C was assumed for the purpose of the calculations. The PVB polymer properties for each zone were defined upon the basis of the tests discussed in Section 2.1.2. As in point 3, no effect of temperature on the mechanical properties of the glass was assumed. Simulations of the head impactor hitting the central point of the windscreen were performed for a set of temperatures as in Table 3.

The character of the HIC curve as a function of temperature was found to be different for the cases of unheated and heated windscreen. In the first case, there is a non-linear relationship between HIC and temperature. The consequence of heating the windscreen was a change to a near linear relationship. The HIC values for the heated windscreen were lower compared to the corresponding temperature levels for the case of unheated windscreen. The value of $HIC_{-30{}^{\circ}C-10{}^{\circ}C}$ = 665 was the highest HIC value for the analysed range of temperatures: −30 to 0 °C.

**Figure 13:**
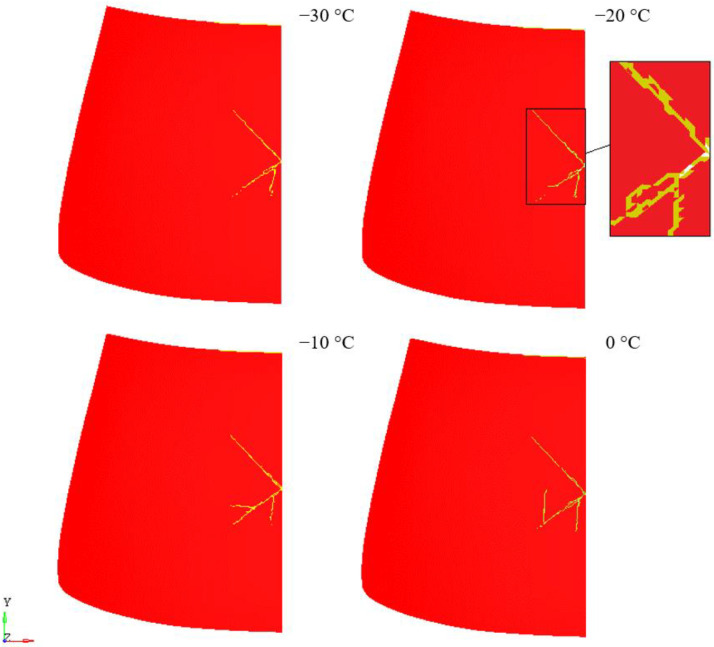
Laminate damage—no heating.

**Figure 14:**
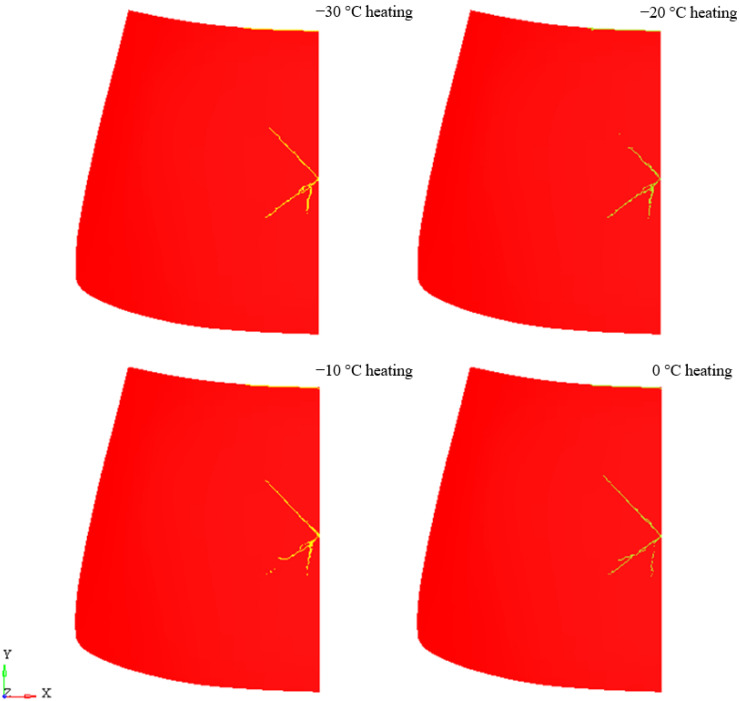
Laminate damage—with heating.

Figure 13 and Figure 14 show the nature of failure of laminated windscreen for the case of unheated and heated windscreen. The obtained results are consistent with the conclusions of the work [43,44,45]. Figure 12 shows the fracture results of laminated windscreen for four temperature cases: 0 °C, −10 °C, −20 °C, and −30 °C. At −20 °C, the rupture of the PVB layer was observed over a small section at the point of pedestrian head impact. For other temperatures there was no rupture of the PVB layer. Looking at the cracking process, we can see a different character for each of the temperatures analysed. The general trend is increase of total length of crack lines with increasing temperature. As the temperature increases, the PVB becomes more susceptible, which implies that the displacement of the glass at the point of impact of the pedestrian head impactor rises—more of the impact energy is transformed into fracturing the glass layers. The exception is the shorter crack line length at −20 °C compared to −30 °C. This is due to the break in the laminate structure (PVB backing layer) at −20 °C.

Figure 13 shows the laminate behaviour for the case of heating the windscreen from the inside for all temperatures analysed. For none of the cases did the PVB layer break. Convergent results were obtained in the analysis of theoretical clusters laminated plates [46,47]. The lowest density of crack lines is at −20 °C, followed by −10 °C. The nature of cracking is different for each temperature, but the differences are no longer as distinct as for cases without heating of the windscreen.

### 4.2. Heating and Fatigue of the Windscreen

The effect of fatigue in laminated windscreen has been determined. The assessment of the effect of fatigue of laminated glass panel has been analysed on the example of a car windscreen, considering loads from torsion of the car body during its operation [48] and consequences of structural changes in the structure of polyvinyl butyral under the influence of external and internal temperature changes (glass heating). The multi-criteria analysis of the pedestrian head impact on the laminated windscreen has been carried out, for which the cases that might occur during the operation of the unit have been taken into account. The functional model of heated laminated windscreen described in Section 4.1. was used for the calculations. Upon the basis of the results of research published in [48], the change in the stiffness of laminated windscreen following the long-term torsional and flexural effects from the operation of the car body has been estimated. The reduction in glass stiffness was estimated at 20% of the original value and related to the glass material data in Table 1. The calculations were based on Maximum tensile strength T = 120 [MPa] and Maximum normalised fractured strength SFmax = 40 [MPa].

The analyses of the head impactor hitting the central point of the windscreen were performed for the same sets of temperatures as described in Section 4.1.

Comparing the HIC values for the cases of windscreen heating and windscreen heating with fatigue, it was found that over the entire temperature range analysed, the safety of VRU would be higher for the glass subjected to operational fatigue. The curve describing the temperature dependence of HIC with fatigue is of a different course and nature to that determined for laminated windscreen with no fatigue loads history. It is a second order curve. The maximum HIC value for the case of heating and fatigue has been obtained for external temperature of −20 °C, i.e., $HIC_{-20\;{}^{\circ}C+0\;{}^{\circ}C+fatigue}$ = 461.7, the minimum value for external temperature of 0 °C, i.e., $HIC_{0\;{}^{\circ}C+20\;{}^{\circ}C+fatigue}$ = 439.

**Figure 15:**
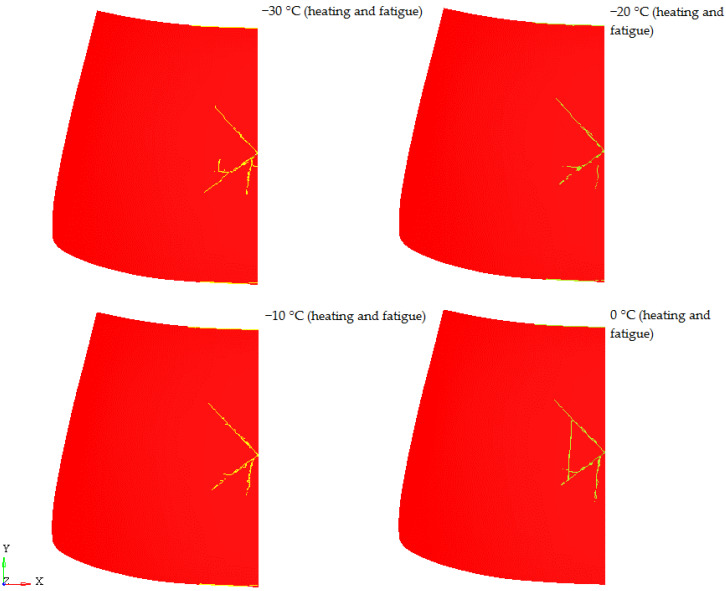
Laminate damage—heating and fatigue.

Figure 15 shows the results of laminated windscreen failure for the case of windscreen heating together with the inclusion of fatigue in the glass layer. Comparing the results for all the temperatures analysed, it was found that the shortest (longest) crack line formed for the component operating at −20 °C, 0 °C, respectively. For none of the cases did the PVB layer break. Comparing the obtained results of fracture of laminated windscreen for the cases of unheated windscreen, heated windscreen, and heated windscreen with fatigue, it was found that the windscreens subjected to fatigue loads originating from torsion of the car body during operation are characterised by the longest glass fracture lines, which is identical as greater energy absorption by the system. The greater energy absorption by laminated windscreen means greater safety of VRU during a vehicle collision.

## 5. Conclusions

The studies of VRU safety in the case of a pedestrian head collision with a laminated windscreen of a passenger car, taking into account the effects of thermo-mechanical loads, allowed for the formulation of conclusions and observations which have not been known and published so far. Starting with the case of an impact in the absence of windscreen heating and comparing the HIC values for all temperatures analysed, it was proven that pedestrian safety significantly decreases with decreasing temperature. Starting at +10 °C, an increase of 43.9% in the HIC maximum was recorded, and at −20 °C the HIC value was 183.5%, compared to the value obtained at the reference temperature of +20 °C.

**Figure 16:**
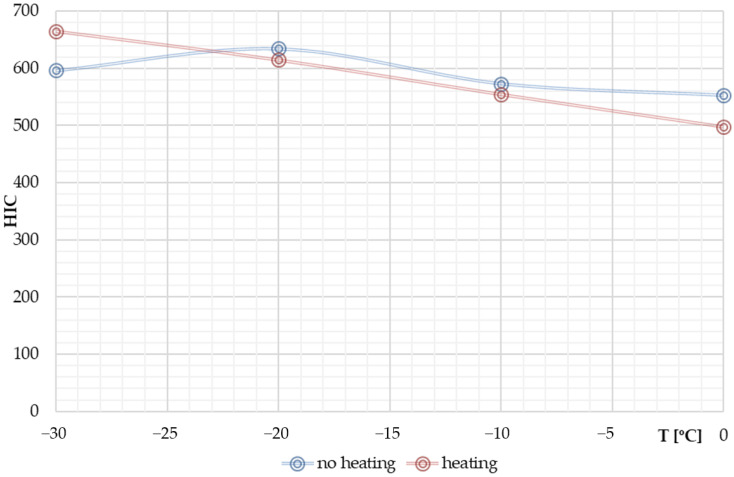
HIC for non-heated and heated windscreen.

Analysing the case of VRU head impact on the laminated windscreen at temperatures below the PVB glass transition temperature, taking into account the heating of the windscreen from the inside by the vehicle heating system, a different character of HIC changes was found than the one observed in the analyses without windscreen heating. HIC for windscreen heating increased as the temperature decreased. Figure 16 shows a comparison of the HIC maxima for the cases: unheated windscreen (blue curve) and heated windscreen (red).

The nature of changes in HIC parameter is linear. ${\mathrm{HIC}}_{-30\;\mathrm{to}\;10{}^{\circ}C}$ was by 11.5% higher than ${\mathrm{HIC}}_{-30{}^{\circ}C}$ = 596, i.e., estimated for the external temperature of −30 °C without windscreen heating. For the external temperature of 0 °C the windscreen heating causes HIC value drop by 9.8%.

**Figure 17:**
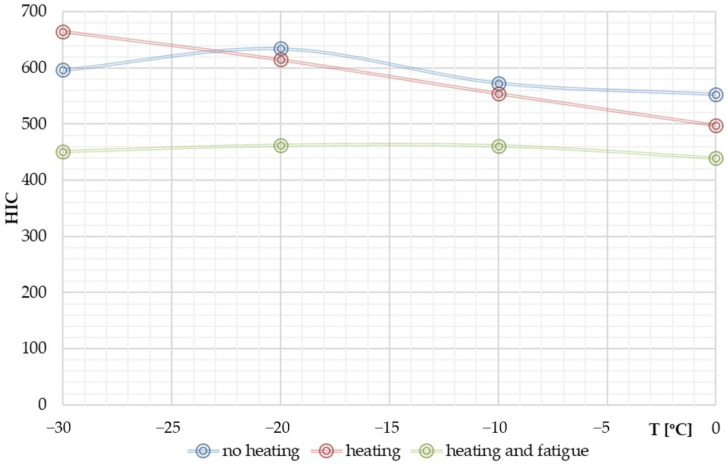
HIC for non-heated windscreen, heated windscreen, and heated windscreen + fatigue.

The effect of the decrease in stiffness of the glass layers following the fatigue loads of the ceramic material on the stiffness of the laminated windscreen and the implications of this on the safety of VRU were determined. Figure 17 shows a comparison of HIC maxima for the case of unheated windscreen, heated windscreen, and heated windscreen with fatigue of the glass layers. It has been proven that the safety of VRU is significantly higher for windscreen subjected to operational fatigue over the entire operating temperature range of the unit.

## Figures and Tables

**Table 1:** Maximum HIC value depending on temperature.

Temperature [°C]	HIC
+20	345.8
+10	497.7
0	553.0
−10	573.4
−20	634.7
−30	596.3

**Table 2:** Figure 11 curves output values.

Glass Transition	Unit	Black Curve	Red Curve
Onset	°C	9.16	8.24
Midpoint	°C	28.44	22.22
Inflect. Pt.	°C	11.23	15.73
Endpoint	mW °C^−1^	24.34	25.28
Infect. Slp.	Jg^−1^ K^−1^	−39.83^−3^	−39.81^−3^
Delta Cp	°C	0.37	0.41
Left Limit	°C	−1.99	0.20
Right Limit	°C∙min^−1^	37.81	35.30
Heating Rate	°C∙min^−1^	10.00	10.00
Result mode sample temp	-	-	-
Midpoint DIN	°C	16.64	16.65
Midpoint ASTM, IEC	°C	16.75	16.76
Midpoint Richardson	°C	22.36	19.99
Delta cp DIN	Jg^−1^ K^−1^	0.39	0.42
Delta cp ASTM, IEC	Jg^−1^ K^−1^	0.15	0.19
Delta cp Richardson	Jg^−1^ K^−1^	79.22 × 10^−3^	0.15

**Table 3:** Division of windscreen zones in relation to temperature.

Temperature of Zone B	Temperature of Zone A
−30 °C	−10 °C
−20 °C	0 °C
−10 °C	+10 °C
0 °C	+20 °C

## Data Availability

The data presented in this study are available on request from the corresponding author. The data are not publicly available due to size of the simulation results.
